# Prevalence and associating factors of atrial fibrillation in patients with hypertension: a nation-wide study

**DOI:** 10.1186/s12872-016-0232-4

**Published:** 2016-03-22

**Authors:** Rungroj Krittayaphong, Ram Rangsin, Bandit Thinkhamrop, Cameron Hurst, Suthee Rattanamongkolgul, Nintita Sripaiboonkij, Ahthit Yindeengam

**Affiliations:** Division of Cardiology, Department of Medicine, Siriraj Hospital, Mahidol University, Bangkok, Thailand; Department of Military and Community Medicine, Phramongkutklao College of Medicine, Bangkok, Thailand; Faculty of Public Health, Khon Kaen University, Khon Kaen, Thailand; Faculty of Medicine, Chulalongkorn University, Bangkok, Thailand; Department of Preventive and Social Medicine, Srinakarinwirot University, Nakornnayok, Thailand; Cancer Registry Unit, Ramathibodi Hospital, Mahidol University, Bangkok, Thailand; Her Majesty cardiac Center, Siriraj Hospital, Mahidol University, Bangkok, Thailand

**Keywords:** Atrial fibrillation, Prevalence, Hypertension

## Abstract

**Background:**

Atrial fibrillation (AF) is a common cardiac arrhythmia and increases risk of ischemic stroke. Data on the prevalence of AF in Thailand is lacking especially in patients with hypertension. The objectives of this study were to determine prevalence of AF in patients with hypertension and to determine factors that are associated with increased prevalence of AF in a multicenter nationwide study.

**Methods:**

A cross-sectional survey for the national outcome evaluation among hypertensive patients visiting 831 public hospitals in Thailand was conducted between 2011 and 2012 to evaluate status of standard care in hypertensive patients visiting public Thailand Ministry of Public Health (MoPH) hospitals. Inclusion criteria were hypertensive patients aged at least 20 years who had received medical care in the targeted hospital for at least 12 months. The main outcome measurement was AF rhythm, and was measured along with potential risk factors age, gender and cardiovascular risk factors.

**Results:**

There were 13207 hypertensive patients who had ECG data recorded during the survey. AF was detected in 457 patients (3.46 %). Prevalence of AF increased with increasing age, was more common in males and in patients with chronic kidney disease (CKD). Multivariable modelling was conducted to assess which factors were most associated with increased prevalence of AF, and the results showed older age followed by male gender, low LDL-cholesterol and increased uric acid levels were the most important risk factors for AF in this population.

**Conclusions:**

Prevalence of AF in hypertensive patients was 3.46 %. Factors associated with increased risk of AF are old age, male gender, low LDL-cholesterol and elevated uric acid level.

## Background

Atrial fibrillation (AF) is the most common sustained cardiac arrhythmia [1, 2]. The prevalence and incidence increases with age and is more prevalent in men than women [2]. It is estimated that the prevalence of AF is approximately 1–2 % [3]. Prevalence of AF in Asian countries has been reported to be slightly lower than in western populations [4–6]. However, due to the much larger population size of Asia, the burden of AF has been estimated to be much larger in the Asian population compared to western countries. It is estimated that approximately 49 million men and 23 million women in Asia will suffer from AF in 2050 [7]. This number is roughly 12 times more than current estimates in United States [8]. AF may be classified as valvular and non-valvular in origin. Majority of AF are non-valvular etiology [3]. Prevalence of AF increases in patients with certain risk factors such as hypertension [9, 10]. Hypertension increases risk of AF by approximately 1.5 fold in both men and women and is likely to be the most important risk factor for developing AF [11].

Non-valvular AF is an important cause of ischemic stroke both in western and Asian population [5, 12]. The annual incidence of ischemic stroke in patients with non-valvular AF has been reported to be around 5 % [5, 12]. There are many risk factors for ischemic stroke in patients with non-valvular AF such as diabetes mellitus, systemic hypertension, old age, heart failure or left ventricular dysfunction, along with a history of ischemic stroke or transient ischemic attack (TIA) [6, 9, 10].

Prevalence of AF in Asian population has been reported from many countries such as China [5, 13], Japan [14], Korea [15], Taiwan [5], and Singapore [16]. There are limited data for the prevalence of AF in Thailand, both in the general population and in patients with risk factors such as hypertension. We sought to determine the prevalence of AF in patients with hypertension among a large nationwide cross-sectional study in patients with hypertension. Secondary objectives of this study were 1) to determine the prevalence of AF in patients with hypertension stratified by age and gender and 2) to determine factors associated with AF.

## Methods

### Study population

A cross-sectional survey for the national outcome evaluation among hypertensive patients visiting public hospitals in Thailand was conducted between 2011 and 2012 to evaluate status of standard care in hypertensive patients visiting public Thailand Ministry of Public Health (MoPH) hospitals in Thailand including private clinics in the Thailand National Health Security Office (NHSO)’s program in Bangkok. Inclusion criteria for the present study were hypertensive patients aged at least 20 years old who received medical care in the participating hospital for the previous 12 months. Patients participating in clinical trials were excluded.

A two-stage stratified cluster, proportional to the size sampling technique was used to select a nationally and provincially representative sample of hypertensive patients in Thailand. For every province outside Bangkok, the targeted hospital included all hospitals that were public hospitals under the MoPH. For Bangkok, the targeted hospital included all hospitals and clinics participating in the Thailand NHSO’s program. The first stage of sampling was at the province level representing 77 strata, and the second stage of sampling was the level of hospital in each province in Thailand. Hospitals in each province were stratified into 5 strata by their sizes i.e., regional center hospital (>500 beds), provincial general hospital (200 – 500 beds), large community hospital (90 – 120 beds), medium community hospital (60 beds), and small community hospital (10 – 30 beds). All university hospitals were excluded from our study.

The study was approved by the Ethical Review Committee for Research in Human Subjects, Thailand Ministry of Public Health, and the Royal Thai Army Medical Department Ethical Review Board as well as local institutional review boards of the participating hospitals. Written informed consent was obtained prior to participation.

### Data collection

There were a total of 831 hospitals under the Thai universal coverage scheme: 25 regional hospitals, 70 general hospitals and 736 community hospitals. All regional and general hospitals were selected as well as 70 % of small community hospital, 20 % of medium size community hospital and 10 % of large community hospital. This faction was based on the proportion of patient care undertaken at the various levels of hospitals. Patients with diagnosis of hypertension were randomly selected according to the proportion of patients registered at each hospital. Sample size of study population was calculated from the proportion to size model for each province.

Data were retrieved from patients’ medical records and included baseline information, status of hypertensive complication, and results of laboratory tests.

### Measurements

Collected variables were demographic data, weight, height, body mass index (BMI), systolic blood pressure (SBP), diastolic blood pressure (DBP), cardiovascular risk factors, blood chemistry data including fasting plasma glucose (FPG), serum creatinine, uric acid, lipid profiles, available ECG data and results, and complication related to hypertension including stroke. Glomerular filtration rate (GFR) was calculated from epidemiology collaboration formula (EPI). Stroke was classified as ischemic stroke, TIA, unspecified stroke, hemorrhagic stroke.

Data management team sent query to study site to verify data if needed. Data and site monitoring was performed in 10 % of study site or approximately 60 hospitals by a random selection process.

ECG is not a prerequisite for the main study. It was noted in the case record form (CRF) whether there were ECG data in the medical record. For those who had ECG, results of the ECG interpretation were recorded. Cardiac rhythm was noted for the presence or absence of AF.

Main outcome measurements include AF rhythm and factors that might be related to AF such as age, gender, cardiovascular risk factors.

### Statistical analysis

Continuous data were presented as mean and standard deviation while categorical variables were presented as number and percentages. Prevalence data was reported as percentage and 95 % confidence interval. Comparisons of continuous data were made by an independent sample *t*-test. For categorical variables, comparisons were made by the chi-square test. Continuous data were grouped for the analysis of Odds ratio as follows; age ≥ 65 years, SBP ≥ 140 mmHg, DBP ≥ 90 mmHg, TC ≥ 200 mg/dl, TG ≥ 200 mg/dl, HDL < 40 mg/dl, LDL ≥ 100 mg/dl, BMI ≥ 25 kg/m2, GFR < 60 ml/min, uric acid ≥ 7 mg/dl in male and ≥ 6 mg/dl in female. Odds ratio and 95 % confidence interval (CI) for univariate analysis were made by the Likelihood ratio test. Multivariate logistic regression analysis (with forward LR) was performed to determine the independent factors associated with increased risk of AF using complete-cases analysis and imputation method for the missing data. Missing values were imputed based on the means of complete cases with noise added based on the t-distribution. A *p*-value less than 0.05 was considered significant. Statistical analysis was performed with SPSS version 20.

## Results

There were a total of 71440 with hypertension enrolled in the main study during 2011–2012. ECG was performed in 13207 patients (18.5 %). Average age was 63.6 ± 11.1 years, 4711 (35.7 %) were male. Baseline variables between patients with and without ECG were numerically similar for SBP (130.8 ± 16.3 vs 129.9 ± 16.0 mmHg), DBP (75.0 ± 10.8 vs 75.4 ± 10.5 mmHg), FPG (116.2 ± 40.4 vs 120.3 ± 43.1 mg/dl), serum creatinine (1.17 ± 0.87 vs 1.17 ± 1.10 mg/dl), total cholesterol (190.7 ± 44.1 vs 192.5 ± 44.2 mg/dl), triglyceride (158.2 ± 92.8 vs 163.7 ± 100.4 mg/dl), HDL-cholesterol (47.8 ± 13.7 vs 48.0 ± 14.3 mg/dl) and LDL-cholesterol (113.5 ± 37.4 vs 114.2 ± 37.3 mg/dl) with a slightly older age for patients who had ECG (63.6 ± 11.1 vs 61.1 ± 11.2 years) and greater proportion of male (35.7 vs 33.7 %).

**Fig. 1:**
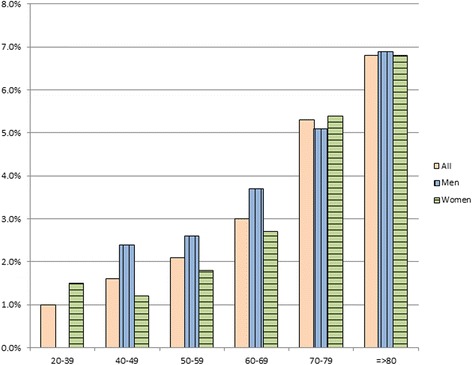
Prevalence of AF in men and women at different age groups

Among 13207 patients with available ECG, AF was detected in 457 patients (3.46 %, 95 % CI 3.16-3.79). Baseline characteristics between patients with and without AF are shown in Table 1. Prevalence of AF increased with increasing age, more common in male and in patients with chronic kidney disease (CKD). Figure 1 showed the bar graph of prevalence of AF for every 10-year of age and separate between men and women. Since the increased prevalence of AF in males was evident only until the 7th decade of life and the prevalence in individuals ≥ 70 years was roughly the same in men and women, we performed additional analysis for interaction between age and gender on the prevalence of AF. The results showed that there was a significant interaction (p for interaction test <0.001) between age and gender on the association of AF.

**Table 1:** Baseline characteristics of patients (total number of subjects = 13207)

Baseline variables	n	Mean ± SD or number (%)
Age	13207	63.6 ± 11.1
Gender	13207	
Male		4711 (35.7)
Female		8796 (64.3)
Current smoker	13207	
yes		750 (5.7)
no		12457 (94.3)
Type of hospital	12800	
Urban		5258 (41.1)
Community		7542 (58.9)
DM	13207	
yes		3653 (27.7)
no		9554 (72.3)
SBP	13197	130.8 ± 16.3
DBP	13186	75.0 ± 10.8
TC	11635	190.7 ± 44.1
TG	11882	158.2 ± 92.8
HDL	11268	47.8 ± 13.7
LDL	11536	113.5 ± 37.4
BMI	11865	25.2 ± 4.49
GFR_EPI	12248	66.7 ± 23.9
Uric acid	5392	6.15 ± 1.95

*SD* standard deviation, *DM* diabetes mellitus, *SBP* systolic blood pressure, *DBP* diastolic blood pressure, *TC* total cholesterol, *TG* triglyceride, *HDL* high density lipoprotein cholesterol, *LDL* low density lipoprotein cholesterol, *BMI* body mass index, *GFR_EPI* glomerular filtration rate by epidemiology collaboration formula

We did not collect the data on antihypertensive medications except for angiotensin converting enzyme inhibitor (ACEI) or angiotensin receptor blocker (ARB) due to the special interest on the use of this drug group in Asian population. ACEI or ARB was prescribed in 2838 patients (21.5 %). There was no significant association between ACEI or ARB and AF (prevalence of AF was 3.0 % in patients with ACEI or ARB and 3.6 % in those without, *p* = 0.157).

**Fig. 2:**
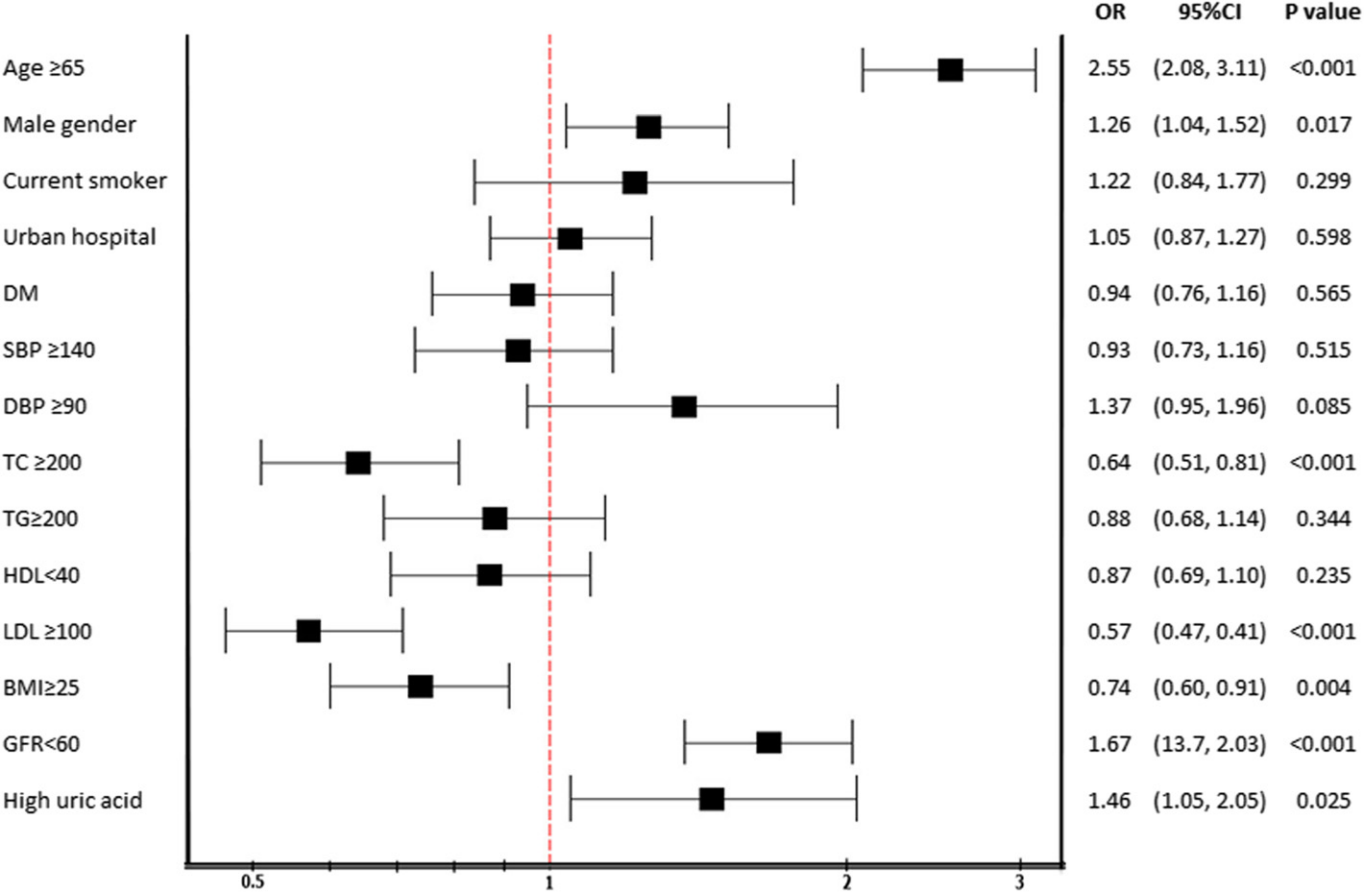
Bivariate analysis factors affecting AF

Figure 2 showed bivariate analysis of demographic data, and cardiovascular risk factors in relation to the prevalence of AF including Odds ratio and 95 % CI. Factors that were associated with increased risk of AF at the *p* value < 0.10 from univariate analysis were old age, male gender, high diastolic blood pressure, low total cholesterol or LDL-cholesterol, normal BMI, impaired renal function, and high uric acid levels. Multivariate analysis of factors that were associated with increased prevalence of AF was performed and the results revealed that the strongest association was old age followed by male gender, low LDL-cholesterol and increased uric acid levels (Table 2).

**Table 2:** Multivariate analysis of factors that had independent association with AF along with the prevalence of AF for complete cases

Factors	%AF	OR_crude_	OR_adj_	95 % CI	OR(IMP)	95%CI
Age ≥65 years	4.1	2.55***	2.86***	1.89–4.33	2.44***	1.99–2.98
Male gender	3.6	1.26*	1.98**	1.35–2.91	0.86	0.71–1.04
LDL <100 mg/dl	4.0	1.74***	1.87**	1.28–2.73	1.62***	1.32–1.96
High uric acid level	3.2	1.46*	1.48*	1.02–2.17	1.26*	1.04–1.51

*p < 0.05, **p < 0.01, ***p < 0.001

*OR*_*adj*_ odds ratio after adjusting for potential confounders, *OR(IMP)* odds ratio after imputating missing data, *CI* confidence interval, *LDL* low density lipoprotein cholesterol

Since a number of patients had missing values for uric acid. This may have decreased the statistical power of the complete-cases analysis. We subsequently applied imputations to handle the missing value and the results of the multivariate analysis after imputation showed that three of the four original independent associations with AF remained significant (Table 2).

Table 3 showed the relationship between number of these 4 factors and the Odds and 95 % CI of AF. The Odds of AF increased from 1.64 in patients with only 1 association factor to 8.99 for those with all 4 factors compared to those without any of these 4 factors.

**Table 3:** Relationship between numbers of independent factors associated with atrial fibrillation and Odds of having atrial fibrillation. Patient without any of these 4 factors were treated as the reference group

Number of factors associated with AF	OR	95 % CI	*P* value
0	reference		
1	1.64	1.07–2.51	0.022
2	2.46	1.66–3.63	<0.001
3	4.30	2.90–6.38	<0.001
4	8.99	5.02–16.07	<0.001

*AF* atrial fibrillation, *OR* Odds ratio, *CI* confidence interval

We also collected the data on the history of ischemic stroke. The prevalence of previous ischemic stroke in this study was 3.8 %. AF was a significant and independent factor associated with ischemic stroke with the OR and 95 % CI of 2.32 (1.64–3.29).

## Discussion

Our study showed that prevalence of AF in Thai population with hypertension was 3.46 %. The prevalence increased in male, elderly, low LDL-cholesterol and high uric acid levels. This is the first report of prevalence of AF in Thai population based on a nationally representative sample. Our study was systematically conducted and employed stratified cluster sampling based on population and hospital size.

AF represents a large burden to the population mainly due to the embolic complication [17]. Ischemic stroke related to AF has been reported to be more disabling than stroke not related to AF [18]. It is one of the most common sustained arrhythmias and more common in the elderly population [1, 2]. Stroke prophylaxis strategies have been shown to be very cost effective [19] although the treatment might cause major bleeding and intracranial bleeding. Anticoagulation is recommended in patients with AF and at least one risk factor [3].

Previous reports indicate that prevalence of AF in western populations is generally greater than Asian populations [4, 6]. There was no clear explanation for this but it could be related to genetic predisposition reported by previous studies on specific polymorphisms that related to AF [20, 21]. We aimed to study prevalence of AF in the Thai hypertensive population since hypertension is a common cardiovascular risk factor and predisposes individuals to AF [11]. Anticoagulant to prevent stroke is recommended in AF patients in the setting of hypertension [3, 18]. Therefore, prevalence of AF in our study may represent an over-estimate in general population, but is likely to be a valid estimate for the Thai hypertensive population. Prevalence of AF in our study was 3.46 % which is greater than 1.21 % and 1.3 % prevalence of AF in general population in Thailand [22, 23], 0.65 % for China [24], 0.56 % for Japan [25] and 0.95 % and 1.2 % prevalence of AF in the United States and United Kingdom [8, 26]. However, our results are comparable with those from a study conducted by Davies and colleagues which reported the prevalence of 5.1 % in male and 2.6 % in female [27].

Univariate analysis of our study showed that factors associated with AF were old age, male gender, low total cholesterol or LDL-cholesterol, normal BMI, impaired renal function, and high uric acid levels. However, after adjusting for confounding, only old age, male gender, low LDL-cholesterol and high uric acid levels remained significantly associated with AF, with elderly as the important factor. Prevalence of AF increases with age due to degenerative process of the atrial muscle and conducting cells [2, 28]. The results of our study are similar with those conducted on both western and other Asian populations [6]. The finding on more predominance in males is also consistent with previous reports [8, 29]. We noticed a significant interaction of age and gender on the prevalence of AF. Male gender was more likely to have AF compared to female in the age group less than 70 years but no such association after the age of 70. This finding was not found in previous reports from Europe [29] and United States [8]. However, similar interaction has been reported from Taiwan [5]. Therefore, the effect of male gender on the increased prevalence of AF in Asian population may disappear after the age of 70 years. Renal impairment has been reported as a risk factor in some previous studies [14, 30]. From our study, however, although renal impairment was associated with AF at the crude level, it did not remain significant when adjusting for other covariates.

We do not have a clear explanation why AF was related to low LDL-cholesterol in our study. Studies about the impact of Hyperlipidemia on AF show mixed results. Indeed, some reports indicate that hyperlipidemic patients have a lower risk of AF [14, 31], as in our study. However, another study showed that hyperlipidemia had a higher risk of AF [24]. The relationship between AF and low LDL-cholesterol in our study should be interpreted with caution due to the cross-sectional nature of our study. It is possible that patients with AF may have dyslipidemia, and treatment with statins was not recorded in our dataset.

For the association we identified between AF and hyperuricemia, there have been few studies to support our findings. Although, some previous studies showed that hyperuricemia is an independent risk factor for AF [32, 33]. It is postulated that hyperuricemia can have effect on the inflammatory and fibrotic process [34] leading to an increased atrial size and eventually AF [32] which may occur with or without a preceding inflammatory process. Some studies showed that treatment of hyperuricemia can prevent inflammatory and fibrosis formation in certain disease such as pericarditis [35, 36].

Results of our study have some clinical implications. Our main objective was to get an accurate estimate of prevalence of AF in hypertensive population. Our prevalence estimate 3.46 % AF in the hypertensive population is high considering the malignant course of this disease that might cause a serious thromboembolic stroke. We demonstrate that there are other factors that could even further increase the prevalence of AF with odds ratio as high as 20 for certain combinations of the associated factors. For example, males older than 65 years of age with an LDL less than 100 mg/dl and elevated uric acid have a very high risk of AF. Regular monitoring with ECG or teaching patients to feel the regularity of their pulse should be considered in practice guideline [3]. Such an approach may help reduce the number of serious strokes related to AF by giving stroke prophylaxis treatment.

There were some limitations of our study. First, this study did not initially plan to study ECG finding, and collection of ECG data was incidental (if it was contained in a patient’s medical record) and only 18.5 % of patients had ECG data. Due to a slightly higher proportion of males and a marginally older sample patient with ECG, compared with those without ECG, the prevalence of AF of 3.46 % may represent an overestimate of prevalence in the Thai hypertensive population. If we adjust for the small difference in age and gender between patients with and without ECG, the prevalence of AF for the whole population (with and without ECG) would be 3.3 % if adjusting for gender, 3.28 % if adjusting for age, and 3.15 % if adjusting for both factors. Secondly, this study did not have data on medications such as antihypertensive medications, antithrombotic medication and statins. Lastly, this study is a cross-sectional study, not a cohort study, so factors associated with AF cannot be concluded to represent risk factors, only factors associated with AF. However, the main strength of this study is that this is s nationwide study and probable one of the first in Southeast Asia region.

## Conclusions

In conclusion, prevalence of AF in hypertensive population in our study was 3.46 % which is higher than prevalence of AF in the general community setting considered in most previous reports. The results from our study are useful for future study of the management of AF.

## Availability of data and materials

The dataset supporting the conclusions of this article is available in http://www.damus.in.th.

## Abbreviations

AF: atrial fibrillation
BMI: body mass index
CI: confidence interval
CKD: chronic kidney disease
CRF: case record form
DBP: diastolic blood pressure
ECG: electrocardiogram
EPI: epidemiology collaboration formula
FPG: fasting plasma glucose
GFR: glomerular filtration rate
HDL: high density lipoprotein
LDL: low density lipoprotein
MoPH: ministry of public health
NHSO: national health security office
SBP: systolic blood pressure
TIA: transient ischemic attack
